# Cochlear implantation under local anesthesia: a possible alternative for elderly patients

**DOI:** 10.1007/s00405-019-05407-7

**Published:** 2019-04-04

**Authors:** Nóra Kecskeméti, Magdolna Szőnyi, Marianna Küstel, Anita Gáborján, László Tamás, Gábor Répássy

**Affiliations:** Department of Otorhinolaryngology, Head and Neck Surgery, Semmelweis University, Szigony utca 36., Budapest, 1083 Hungary

**Keywords:** Cochlear implantation, Ageing, Local anesthesia, Elderly patients, Posterior suprameatal approach

## Abstract

**Introduction:**

As average life-expectancy increases, a sufficient hearing rehabilitation for elderly patients with severe-to-profound sensorineural hearing loss becomes more important. Cochlear implantation is a relatively safe surgical procedure also for elderly patients, the higher risk is caused by general anesthesia. We report on four patients who underwent cochlear implantation under local anesthesia.

**Methods:**

After detailed preoperative examinations (audiological tests, imaging, genetic tests, evaluation of motivation and compliance of the patient), four patient with severe-to-profound hearing loss were selected for cochlear implantation under local anesthesia. For the electrode insertion, we used the posterior suprameatal approach technique. Pre- and postoperative pure tone audiometry and speech-perception tests were conducted to prove the success of the procedure.

**Results:**

The mentioned technique was applied; the average length of the operation was 52 min. The intraoperative measurements showed normal impedance and normal neuronal response telemetry, all the patients had sound experience during the intraoperative examination of the engineer. No complications were observed. The postoperative audiological tests showed a significant increase in the hearing perception.

**Conclusion:**

Cochlear implantation under local anesthesia is a safe and fast procedure for elderly patients. The intraoperative sound experience can give an extra motivation in the postoperative rehabilitation. Our results prove that by carefully selected elderly patients cochlear implantation can assure a significant increase in speech perception. We can establish that the new posterior suprameatal approach technique combined with local anesthesia presents a viable future option for those patients who were inoperable beforehand because of high risks of general anesthesia.

## Introduction

According to a statement of the World Health Organization (WHO) published in March 2018, there are 466 million people worldwide with hearing impairment. While 34 million of them are children, one-third of the elderly population (i.e., people over 65 years of age) is affected by this disease. Twelve percent of the world’s whole population is older than 60 years, which ratio will reach 22% by 2050 [1]. This elderly age group is characterized by decreased learning aptitude, social isolation and decreased ability to care for themselves. Depression is also more common among elderly people, the incidence in Hungary being 43% in male and 54% in female patients over 64 years of age [2]. These ratios are even more pronounced among the hearing-impaired. As average life-expectancy increases, sufficient hearing rehabilitation of elderly patients becomes more important.

The most successful type of rehabilitation in severe-to-profound sensorineural hearing loss is cochlear implantation. The wide range of indication (pre- or postlingual, uni- or bilateral, severe to profound sensorineural hearing loss (SNHL) [3]) provides basis for rehabilitation also in the elderly patients. Unlike natural hearing, the cochlear implant uses digital, electronic signals, demanding adequate cognitive function, which is an important factor during the patient selection procedure.

Cochlear implantation is a relatively safe procedure also for elderly patients [4]; the higher surgical risk is caused by general anesthesia.

### Risks of general anesthesia in the elderly

During preoperative risk assessment, it is important to conduct a detailed medical examination. The different associated co-morbidities increase surgical risk and poly-medication can result in drug interactions [5]. It should be highlighted that the pharmacokinetic and pharmacodynamic characteristics are changing with the age of the patient. Reduction of dosage should be considered to avoid a toxic damage to these patients [6]. It is important to stay clear of medication which causes deep sedation during the operation as their more pronounced effect on the central nervous and cardio-respiratory system increases the risk of side-effects such as anterograde amnesia, respiratory deprivation or hypotonia [6]. In the postoperative period, the risk of thromboembolic complications is higher. In the early postoperative period, the incidence of postoperative delirium (POD) is 15% among patients above the age of 60 [7]. In POD, a cognitive dysfunction appears (such as disturbance of cognitive functions and memory, disorientation) which leads to elongated hospitalization. A long-lasting complication of general anesthesia can be postoperative cognitive dysfunction (POCD) [7] which appears as slowed psycho-motor function, mild change in personality which can last for weeks or months. Both POD and POCD can postpone the start of rehabilitation after a cochlear implantation and influence the efficiency of the procedure.

### Cochlear implantation under local anesthesia

Toner et al. [8] published the first cochlear implantation under local anesthesia in 1998. A few other articles supporting the safety of this procedure were also published since then [9–11]. The fundamental conditions of this procedure are the short operating time and the cooperation of the patient. It is also notable that it is often possible to immediately evoke sound sensation during the intraoperative measurements after a successful implantation. To minimize the operation length, different surgical techniques have been developed in recent years. Toner et al. [8] preferred the posterior tympanotomy. Later on the focus has been changed to the suprameatal approach technique [10–12]. In this approach, there is no need to drill the whole mastoid, only a narrow tunnel is created above the outer ear canal, which results in a shorter surgical procedure length. Our working group has developed a modified technique and named it posterior suprameatal approach (PSMA), we have now been performing PSMA for more than 10 years [13]. The surgical method depends on the preferred technique, the mastoidectomy with posterior tympanotomy approach could be also routinely used under local anesthesia. The advantage of PSMA procedure is that the tunnel ends behind the long process of the incus in the tympanic cavity which results in a lower risk of injury of the incus and the facial nerve and avoids removing a part of the annulus which also results in a shorter surgical procedure length.

## Methods

Since February 2016, we implanted 4 patients under local anesthesia in the Department of Otorhinolaryngology Head and Neck Surgery of Semmelweis University.

Detailed preoperative investigation such as medical evaluation, audiological tests (pure tone audiometry, speech-perception tests, ABR-ASSR, otoacoustic emission), imaging (CT, MRI) and also genetical tests (*GJB2* gene sequence analysis) were conducted. The motivation and ability of cooperation were also considered during the patient selection.

Preoperatively, 7.5 mg midazolam was given orally. The local anesthesia was applied with an injection solution of 20 mg lidocaine and 0.01 mg epinephrine per milliliter in the external ear canal under the skin of the superior and posterior wall and doubly diluted in the retroauricular region on the mastoid plane from the upper edge of the auricle until the mastoid tip, but the facial nerve should not be anesthetized. We also inject this solution near the planned bed of the cochlear implant. We used the posterior suprameatal approach in all cases. Detailed description of this method was published [13]; here we briefly summarize this technique.

Epithelium of the auditory canal was detached, the tympanic ring and membrane were elevated, and a lateral tympanotomy was performed.

**Fig. 1:**
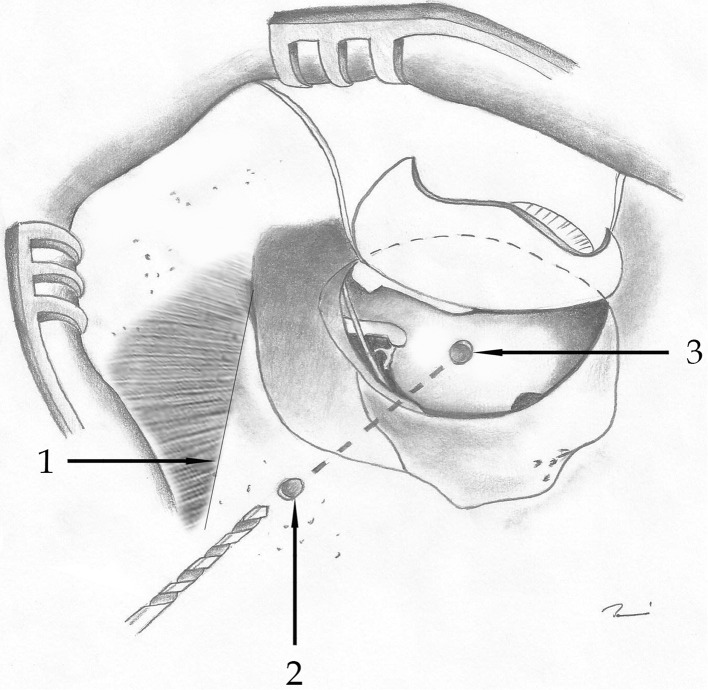
The posterior suprameatal approach. (1) Linea temporalis, (2) beginning of the tunnel, (3) position of the cochleostomy. The canal ends behind the long process of the incus and avoids removing a part of the annulus. (The figure is published with the permission of all authors of the original article [13].)

Cochleostomy was implemented by drilling the promontory using a 0.8–1.0 mm burr. Drilling of the posterior suprameatal bone tunnel was 8 mm posterior to the bony auditory canal, directed toward the promontory (twist drill bit, AESCULAP Twist Drill D2.0-mm L65/35-mm round shaft; AESCULAP AG; Tuttlingen, Germany). The bony tunnel was expected to reach the tympanic cavity medial to the chorda tympani. The creation of a shallow “bone bed” and a pit for the electrode was conducted. The active electrode was inserted into the tympanic cavity through the posterior suprameatal tunnel and then further into the cochlea through the cochleostomy to the scala tympani (Fig. 1).

There was no substantial difference from the technique under general anesthesia. Communication with the patients during surgery is complicated, lipreading and short written comments were used. Patients were informed beforehand about possible dizziness or pain, they must keep their head still and elevate their hand instead. Patients did not demonstrate any pain, they were also asked about pain and discomfort after surgery, but bad experience was not mentioned, neither was any taste disturbance reported. Impedance tests and neural response telemetry (NRT) measurement were carried out intraoperative. NRT measurements were started in low levels and gradually elevated until the patient indicated sound experience, then the next channel was measured. 80 Hz stimulus presentation frequency was used during NRT measurements, because this helps reduce loudness perception so that current level that may be required can be increased. After a long-term usage of the processor, a control pure-tone audiometry tests were conducted.

## Results

Four patients were selected at our institute: 2 males and 2 females. The mean age was 80.7 years (73–86 years) (Table 1). The first patient (P1) suffered from a progressive, bilateral, moderately severe to profound sensorineural hearing loss. Co-morbidities were hypertonia and generalized atherosclerosis. P2 and P3 had progressive bilateral otosclerosis. P2 had ischemic heart disorder, arrhythmia and pacemaker implantation before. P4 had acute unilateral profound hearing loss on the right side due to occlusion of the right anterior inferior cerebellar artery. This patient had metabolic syndrome and asthma bronchial. None of our patients had genetic alterations in the *GJB2* gene.

**Table 1:** The four cases with cause and type of hearing loss, co-morbidities and the result of the pre- and postoperative speech perception tests

Patient	Age	Cause of hearing loss	Severity of hearing loss	Co-morbidities	ASA score [14]	Speech perception preoperative^a^ (%)	Speech perception with CI^a^ (%)
P1	81	Progressive bilateral SNHL circulation disorder	Progressive bilateral moderately severe-profound SNHL	Hypertension, generalized arteriosclerosis	III	0	40
P2	86	Progressive bilateral otosclerosis, right side stapedectomy with acute profound hearing loss in 1985	Bilateral severe–profound SNHL	Ischaemic heart disorder, arrythmia, pacemaker implantation	III	0	60
P3	83	Progressive bilateral otosclerosis	Bilateral profound hearing loss	Hypertonia	II/III	0	45
P4	73	AICA thrombosis on the right side	Acute profound SNHL right side	Metabolic syndrome, bronchial asthma	III	100	100

All patients belonged to a higher risk category for general anesthesia based on the ASA classification score

*ASA* American Society of Anesthesiologists, *AICA* anterior inferior cerebellar artery, *CI* cochlear implant, *P* patient, *SNHL* sensorineural hearing loss

^a^The freefield monosyllabic word recognition test

In all four cases, a Nucleus CI522 electrode was applied and the whole active part of the electrode was implanted. The mean length of the surgical procedure was 52 min (45–60 min). During the intraoperative measurements, the electrodes showed normal impedance and all patients had sound experience during NRT examination of the acoustical engineer. No complications were observed during the postoperative period. The postoperative audiological tests in three patients (P1, P2, and P3) show a significant improvement in the pure tone audiometry and the speech perception as well. Freefield monosyllabic word recognition test was used; validated sentence tests are not available in Hungarian language. P4 has single-sided deafness due to acute profound NSHL, troublesome tinnitus and dizziness. Her tinnitus and dizziness were not improved by the applied systemic and intratympanic corticosteroid treatment, but after the implantation and the setup of the speech processor these symptoms were diminished so her quality of life improved. In case of P1, P2, and P3 communication using high-performance hearing aid was not possible, but after implantation our patients and their families were satisfied with the improved speech perception (Table 1).

## Discussion

Safe surgical procedures with minimal risks are gaining a more important role in the aging population. Hearing is one of the most important senses in daily communication, and elderly people could easily get socially isolated due to their hearing loss.

In this study, we could demonstrate that cochlear implantation under local anesthesia can be a safe alternative of procedures under general anesthesia.

Possible surgical approaches are mastoidectomy with posterior tympanotomy or suprameatal approach. Few articles are available about cochlear implantation under local anesthesia. A great majority of the patients are operated under general anesthesia. Sometimes elderly patients show up with profound hearing loss and with a lot of other medical problems. For these elderly people (above 80 years old), general anesthesia may result in undesirable situations, for example permanent or definitive deterioration of their condition. This will influence not only the physical status but mental health as well. For these reasons, it is very important to find safe solutions for their rehabilitation. Elderly people are afraid of general anesthesia but as our practice shows, they easily accept some possibility of pain or sudden dizziness after detailed explanation of the procedure. In adequate local anesthesia with some per oral sedation they did not feel any pain and were absolutely satisfied with the method. During our operation they were able to communicate.

The local anesthesia for elderly patients is often more feasible than general anesthesia; Mistry et al. showed that patient satisfaction is also relatively high [15]. POD and POCD could be avoided thanks to this method which also shortens the time of hospitalization and leads to earlier rehabilitation. However, precise patient selection is of utmost importance. The local anesthesia requires a cooperative and motivated patient, and the cognitive functions are also important while adjusting to the new type of hearing. Selecting the right patients might require a longer preoperative examination period, but the more thorough the patient selection procedure is, the greater the chance for successful rehabilitation can be.

The used posterior suprameatal approach is a new technique in cochlear implantation [13]. The procedure can be considered safe due to lower risk of injury to the incus and the facial nerve and allows a shorter operating time. Using other techniques cochlear implantation is also a routinely applied rehabilitation, but duration of the operation may be longer. In these cases, application of anesthetic products with prolonged effectiveness, e.g., bupivacaine (Marcaine) or intraoperative light sedation (propofol) or the use of intravenous analgetics (fentanyl) may be useful tools. In the latter situation measurement of the oxygen saturation, blood pressure, etc., and presence of an anesthetist is necessary. Sometimes the effect on the mental function and interaction of these drugs are not precisely predictable, so we avoided using them.

It was described previously that the quality of life in the elderly increased after cochlear implantation [16]. The results of our postoperative examinations showed that these procedures were exceedingly successful. We observed improvement in the pure-tone audiometry and the speech-perception tests in all cases. Their tinnitus became milder and the vertigo and instability disappeared. It seemed rightful to conclude that the quality of life of our patients improved.

Considering the economic aspects, Shabashev et al. [17] showed that cochlear implantation under local anesthesia also displayed lower costs and increased economic benefits due to prolonging independence and reducing the incidence of dementia. Shorter surgical times and less medication can reduce the length of hospitalization. All of these factors are very important in cost-effectiveness. However, to prove this theory, further cost-effectiveness studies in a larger patient population would be needed.

Finally, we concluded that the posterior suprameatal approach combined with local anesthesia presented a viable future option for surgical hearing rehabilitation for elderly patients who were inoperable beforehand because of high risk associated with general anesthesia.
